# Effect of Remimazolam on the Incidence of Intraoperative Hypothermia Compared with Inhalation Anesthetics in Patients Undergoing Endoscopic Nasal Surgery: A Prospective Randomized Controlled Trial

**DOI:** 10.7150/ijms.100262

**Published:** 2024-09-30

**Authors:** Sung-Ae Cho, Seok-Jin Lee, Woojin Kwon, Ji-Yoon Jung, Hwang-Ju You, Si-eun Yoon, Tae-Yun Sung

**Affiliations:** 1 Department of Anaesthesiology and Pain Medicine, Konyang University Hospital, Konyang University Myunggok Medical Research Institute, Konyang University College of Medicine, Daejeon, Republic of Korea.; 2 Department of Anaesthesiology and Pain Medicine, Konyang University Hospital, Konyang University College of Medicine, Daejeon, Republic of Korea.

**Keywords:** Remimazolam, Hypothermia, Nasal Surgical Procedures, Desflurane

## Abstract

Background: Remimazolam is an ultrashort-acting benzodiazepine that is increasingly used for its efficacy in anesthesia induction and maintenance. However, limited research has explored its impact on intraoperative hypothermia compared to that of traditional inhalation anesthetics. This study aimed to compare the incidence of hypothermia during endoscopic nasal surgery when using remimazolam for maintenance anesthesia versus using inhalation anesthetics.

Methods: This prospective study included 70 patients who underwent endoscopic nasal surgery under general anesthesia. The patients were randomly assigned to one of two groups: the inhalation anesthetic (IA) group (n=35), in which desflurane and nitrous oxide were administered, and the remimazolam (R) group (n=35), in which remimazolam and remifentanil were administered for anesthesia maintenance. The primary outcome was the incidence of intraoperative hypothermia, defined as an esophageal temperature below 36 °C during anesthesia.

Results: The incidence of intraoperative hypothermia was significantly higher in the R group than in the IA group (P = 0.014). Furthermore, the temperature at the end of the surgery was significantly lower in the R group than in the IA group (P = 0.006). Additionally, the use of warming devices after surgery was more frequent in the R group than in the IA group (P = 0.047).

Conclusions: These findings suggest that the use of remimazolam for maintenance anesthesia during endoscopic nasal surgery increases the risk of intraoperative hypothermia compared to the use of inhalation anesthetics. This highlights the importance of temperature monitoring in patients receiving remimazolam to minimize the adverse outcomes associated with hypothermia during surgery.

## Introduction

Perioperative hypothermia is a common complication of general anesthesia, with reported incidence rates ranging from 50% to 90% 1. This complication can lead to various adverse outcomes, including surgical site infections, prolonged hospital stays, cardiac events, coagulation disorders, and compromised immune responses 2-4. Most general anesthetics impair thermoregulatory control, resulting in reduced vasoconstriction and shivering thresholds 5. Additionally, anesthetized patients lack a behavioral response to shivering, which further contributes to the challenge of maintaining a normal body temperature during surgery. While various anesthesia societies worldwide recommend temperature monitoring during surgical procedures, and different modalities are employed to prevent intraoperative hypothermia, the optimal agent for or approach to prevention remains unidentified 2, 6-9.

Desflurane is a general anesthetic commonly used for maintenance during surgery. However, because of its pungent characteristics, it usually requires another induction agent, such as propofol. The combination of propofol for induction and desflurane for maintenance is commonly used for general anesthesia; however, the specific thermoregulatory effects of this combination are not well established 10, 11. In contrast, remimazolam is a novel ultrashort-acting intravenous benzodiazepine that can be used both as an induction and maintenance agent for general anesthesia, and is known for its hemodynamic stability and lack of injection pain. Moreover, midazolam, another benzodiazepine, causes minimal impairment of thermoregulatory control, while remimazolam has been confirmed to affect thermoregulatory vasoconstriction thresholds 12.

Studies on the thermoregulatory effects of remimazolam are scarce. Those that have been conducted have reported that remimazolam is superior to other anesthetics such as propofol or sevoflurane in maintaining body temperature during anesthesia in patients undergoing prostate and gynecological surgery 12, 13. However, these studies may have been influenced by confounding factors, such as the type of surgery, patient positioning (e.g., lithotomy position), intraabdominal cold carbon dioxide insufflation, and the relatively large amount of intraoperative fluid administration and blood loss, which could affect comparisons of anesthetic effects on body temperature. It is difficult to find studies on body temperature during surgery in which patient position and surgical characteristics may have relatively little effect thereon. Furthermore, to the best of our knowledge, there are no studies that compare the effects of desflurane and remimazolam on intraoperative hypothermia. Therefore, this study aimed to investigate and compare the incidence of intraoperative hypothermia in patients receiving remimazolam and desflurane as anesthesia maintenance agents for endoscopic nasal surgery.

## Materials and Methods

### Study Design and Patient Selection

This prospective randomized controlled trial was conducted between November 2021 and August 2023 at a single university hospital. The study was approved by the Institutional Review Board (KYUH 2021-09-021) and registered with the Korean Clinical Research Information Service (https://cris.nih.go.kr/, KCT0006838). Written informed consent was obtained from the participants or their legal guardians.

Patients aged 19-65 years who underwent endoscopic nasal surgery under general anesthesia and had an American Society of Anesthesiologists physical status (ASA PS) of I-II were included. The exclusion criteria were as follows: preoperative body temperature ≥ 37.6 °C or < 36.0 °C; obesity (body mass index > 35 kg/m^2^); severe endocrine, cardiovascular, or metabolic disease (determined by medical consultation); hemodynamic instability; respiratory failure; history of neuropsychiatric disease or cognitive disorder; contraindications to remimazolam (e.g., hypersensitivity to benzodiazepine drugs, glaucoma, alcohol or drug dependence, sleep apnea syndrome, renal failure, liver failure); anesthesia duration < 1 h or > 3 h; and emergency surgery.

Patients were randomly assigned to either the inhalation anesthetics group (IA group) or the remimazolam group (R group) in a 1:1 ratio using online randomization software (Research Randomizer; www.randomizer.org). All patients received the same anesthetic protocol except for the anesthesia maintenance agent. Anesthesia procedures were conducted by an anesthesiologist who was not involved in the data collection.

### Anesthesia

All patients arrived in the operating room after fasting for a minimum of 8 h. The preoperative holding area and post-anesthesia care unit (PACU) was maintained at an ambient temperature of 22 to 25 °C, while the operating room was kept at 21 to 24 °C. Upon arrival at the preoperative holding area, the patients' baseline temperature was measured using an infrared tympanic membrane thermometer (Thermoscan IRT 4020; Braun GmbH, Kronberg, Germany), and the highest temperature obtained from both ears was recorded.

Routine monitoring, including electrocardiography, noninvasive blood pressure measurement, pulse oximetry, bispectral index (BIS), and neuromuscular monitoring, were initiated in the operating room. The systolic blood pressure (SBP) and heart rate (HR) that were measured before anesthesia induction were considered the baseline values. For the induction of anesthesia, both groups received propofol (2 mg/kg), fentanyl (1 mcg/kg), and rocuronium (0.6 mg/kg) intravenously. The respiratory rate of controlled mechanical ventilation was adjusted to maintain an end-tidal carbon dioxide concentration of 30 to 35 mmHg during surgery. In the IA group, anesthesia was maintained with desflurane and 50% nitrous oxide (0.8-1.2 of age-adjusted minimum alveolar concentration [MAC]) to maintain a BIS of 40 to 60. In the R group, anesthesia was maintained with continuous intravenous infusion of remimazolam (1-2 mg/kg/h) and a target-controlled infusion of remifentanil (effect-site concentration, 2 ng/mL based on Minto's conceptual model) to sustain a BIS of 40 to 60.

After the induction of general anesthesia, an esophageal thermistor probe (L000412, Gonimed Co., South Korea) was inserted at the point where the heart sound was the loudest on auscultation. The intraoperative core temperature, SBP, and HR were measured every 15 min until the end of surgery. A forced-air warming blanket (Bair Hugger^TM^ Lower-Body Cover Model 52500, Heater Model 505; Arizant Healthcare Inc., USA) was used to cover the body below the xiphoid process.

Throughout anesthesia, inhaled gas was supplied through a respiratory circuit heated to 39.5 °C and humidified, and intravenous and irrigation fluids were administered at room temperature. Following anesthesia, the volumes of intravenous and irrigation fluids and the estimated blood loss were recorded by the surgeon.

In the PACU, forced-air warming with a set temperature of 43 °C was applied if the patient reported feeling cold, if the tympanic temperature was < 36 °C, or when shivering was observed. In case of shivering, meperidine 25 mg was administered intravenously. Postoperative pain was assessed using a numerical rating scale (NRS; 0 = no pain, 10 = worst imaginable pain). If the NRS score exceeded 4 and analgesics were requested, fentanyl 0.5-1 μg/kg was administered intravenously. All adverse events (e.g., nausea, vomiting, headache, dizziness, dyspnea, desaturation, and confusion) were recorded meticulously.

### Outcome Measures

Intraoperative hypothermia was defined as an esophageal temperature < 36 °C during anesthesia, and the severity of hypothermia was evaluated as mild (35.5-35.9 °C), moderate (35.0-35.4 °C), or severe (34.5-34.9 °C) 14. If the esophageal temperature measured during anesthesia decreased below 36 °C even once, it was considered intraoperative hypothermia. The primary outcome was the incidence of intraoperative hypothermia in the IA and R groups. The secondary outcomes were the severity of intraoperative hypothermia, core temperature at the end of surgery, incidence of shivering, number of patients receiving active warming in the PACU, core temperature changes during the first hour after anesthesia induction, and hemodynamic changes during the first hour after anesthesia induction. All outcome variables were collected by anesthesiology residents who were unaware of the purpose of this study and were not involved in patient care.

### Statistical Analysis

In a preliminary study (n=20 per group), the incidences of intraoperative hypothermia were 65% (13/20) and 30% (6/20) in the R and IA group, respectively. When the sample size was calculated with a power of 0.8, two-tailed α value of 0.05, and 1:1 allocation ratio using G*Power software (version 3.1.9.7; Franz Faul, Universitat Kiel, Germany), it was found that 31 patients were required per group. Therefore, considering a potential dropout rate of 10%, 35 patients were included in each group.

After assessing the data distribution using the Kolmogorov-Smirnov test, Student's *t*- or the Mann-Whitney U test was used to compare continuous variables, which are presented as mean ± standard deviation or median (interquartile range). The χ2 test, χ2 test for trends (linear-by-linear association), or Fisher's exact test were used to compare categorical variables, which are expressed as percentages (%) or numbers. Variables measured repeatedly over time, such as temperature, SBP, and HR, were analyzed using repeated-measures analysis of variance (ANOVA) with Bonferroni correction. Statistical significance was set at P < 0.05 for all analyses. Statistical analyses were performed using IBM SPSS Statistics for Windows (version 27.0; IBM Corp., Armonk, NY).

## Results

**Figure 1:**
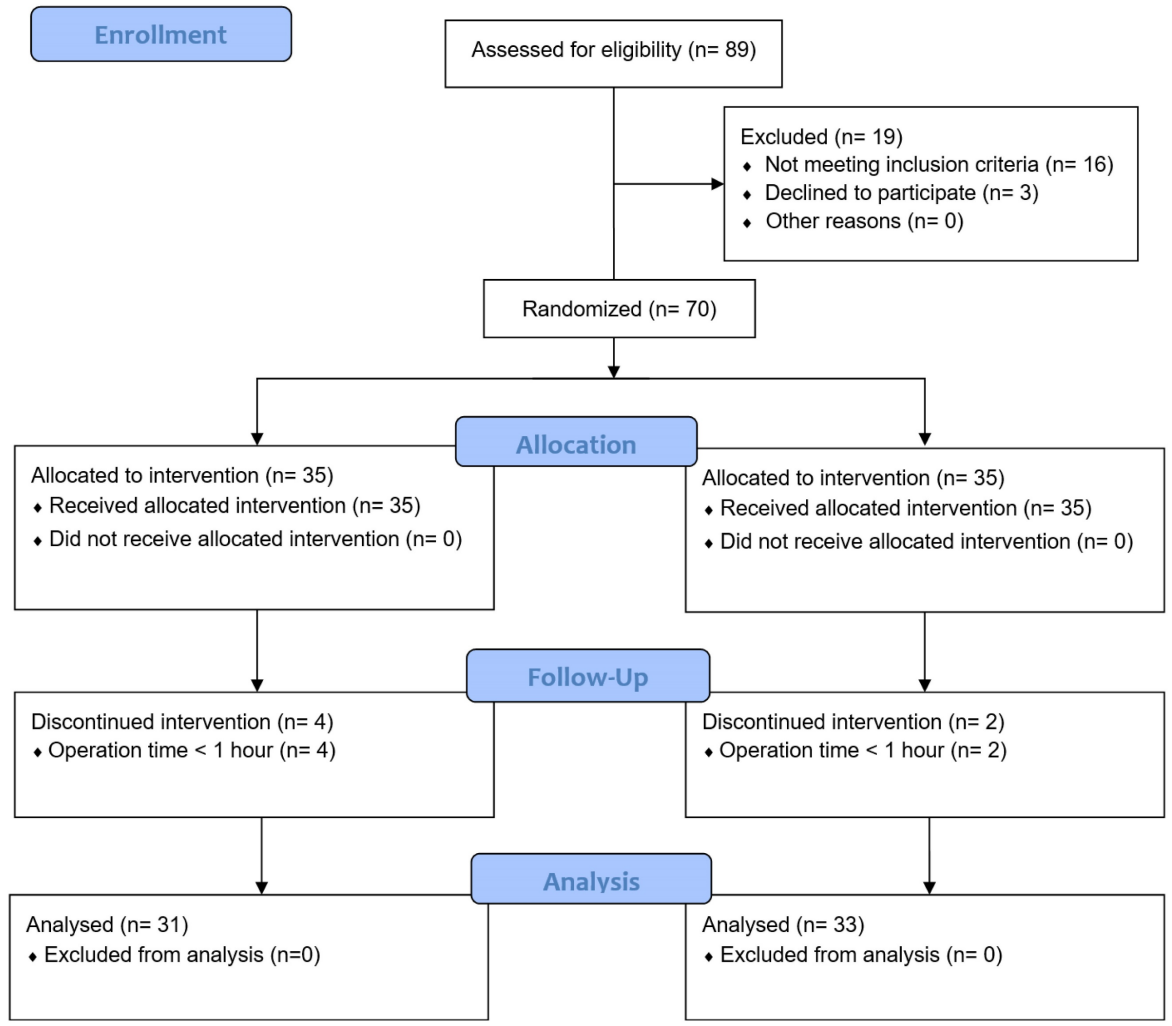
Consort diagram

Initially, 89 patients were considered eligible. However, 19 patients did not meet the inclusion criteria; thus, 70 patients were enrolled and randomly assigned to either the IA or R group. Six patients were excluded because their operating times were < 1 h. Consequently, 64 patients (31 in the IA group and 33 in the R group) were finally included in the study (Figure 1). The baseline data and patient characteristics were comparable between the two groups (Table 1).

Table 2 shows details on intraoperative temperatures and hypothermia. The R group exhibited a significantly higher incidence of intraoperative hypothermia compared to the IA group (63.6% [21/33] vs. 32.3% [10/31]; relative risk [RR] 1.863; 95% confidence interval [CI] for RR 1.16 to 3.110; effect size h = 0.637; P = 0.014). Additionally, the temperature at the end of surgery was significantly lower in the R group compared to the IA group (35.8 ± 0.6 °C vs. 36.3 ± 0.4 °C; mean difference [MD], P = 0.240, 95% CI for MD 0.071 to 0.408; effect size d = 0.845; P = 0.006). The thermal comfort score in the PACU, incidence of shivering, and shivering grade distribution were comparable between the two groups. However, the incidence of the use of a warming device was significantly higher in the R group than in the IA group (33.3% vs. 9.7%, P = 0.047).

**Figure 2:**
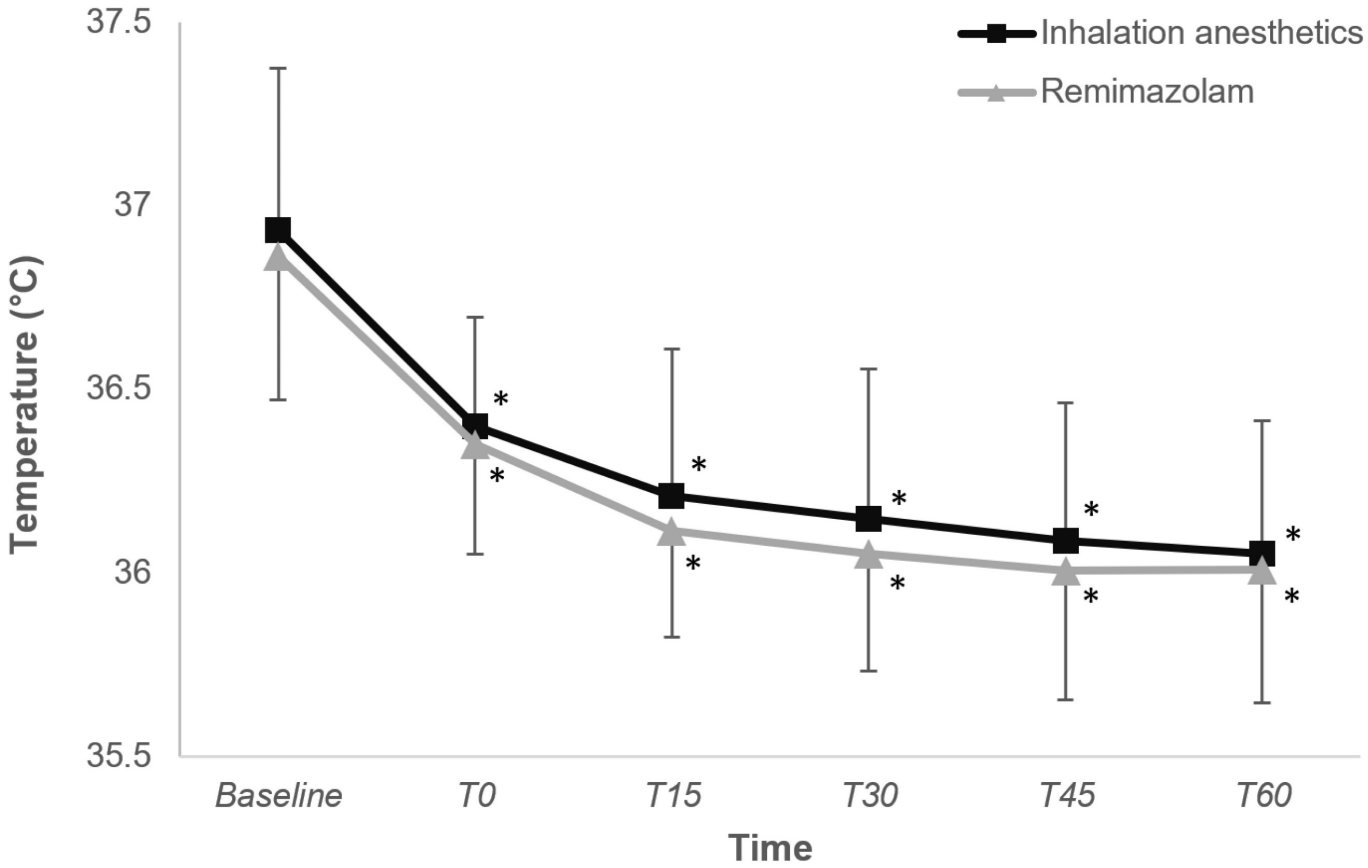
Change in core temperature during intraoperative period in the inhalation anesthetics and remimazolam group. *P < 0.05, vs. baseline in each group (Bonferroni-corrected). T0-T60, 0-60 minutes after induction of anesthesia.

Figure 2 depicts the changes in core temperature within the first hour of anesthesia induction, revealing a comparable trend between the two groups (P = 0.425).

**Figure 3:**
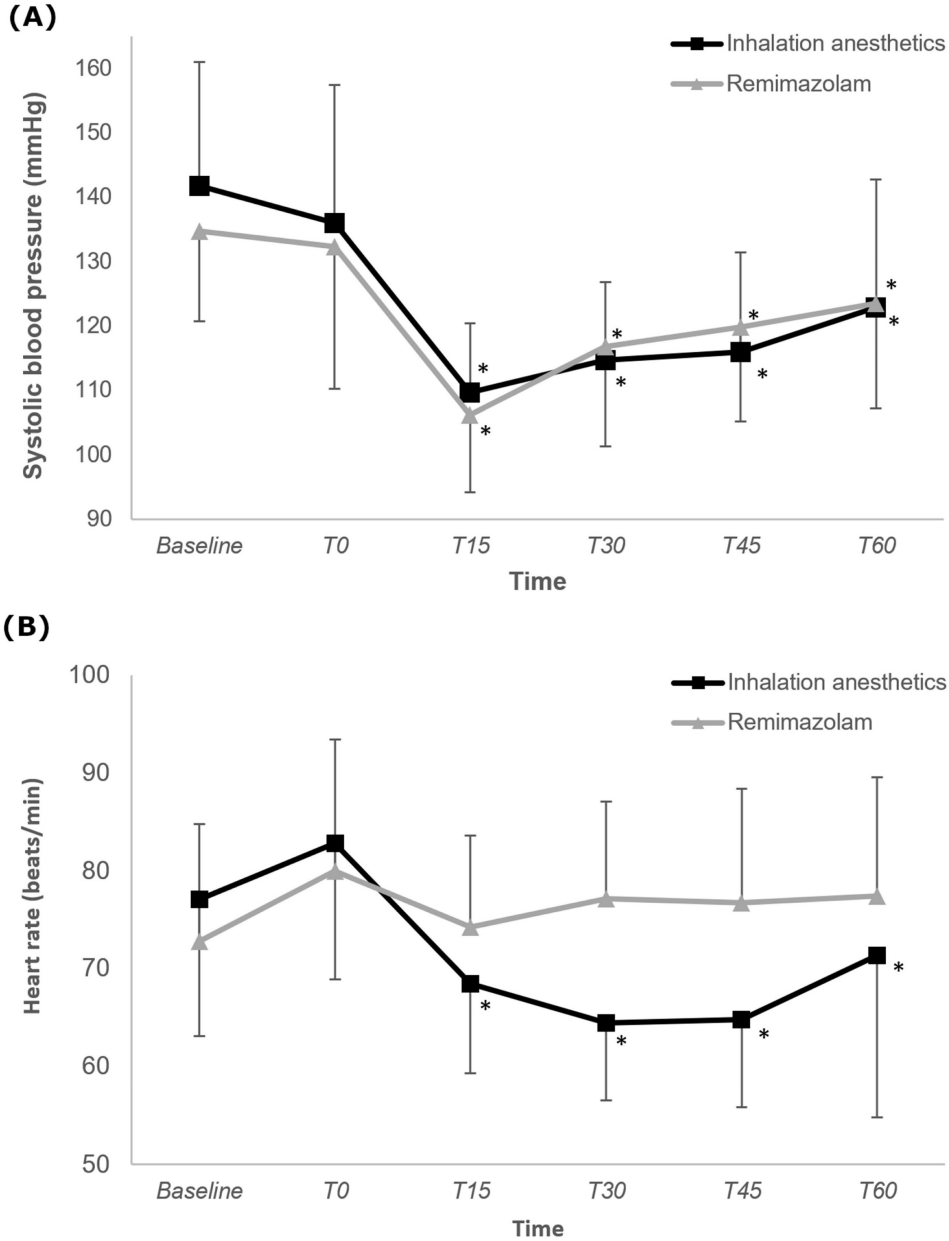
Change in systolic blood pressure and heart rate during intraoperative period in the inhalation anesthetics and remimazolam group. *P < 0.05, vs. baseline in each group (Bonferroni-corrected). T0-T60: 0-60 minutes after induction of anesthesia.

Figure 3 illustrates changes in SBP and HR. The changes in SBP did not differ significantly between the two groups (P = 0.201), with both groups showing a significantly lower SBP at 15, 30, 45, and 60 min after anesthesia induction than at baseline (Bonferroni-corrected P < 0.05). The trend in HR change significantly differed between the two groups (P < 0.001), indicating a consistent HR in the R group. Only in the IA group was the HRs at 15, 30, 45, and 60 min after anesthesia induction significantly lower than the baseline HR was.

Table 3 shows the postoperative data and adverse events. There were no significant differences between the groups.

## Discussion

This study evaluated the impact of remimazolam and inhalation anesthetics as maintenance agents on intraoperative hypothermia in patients undergoing endoscopic nasal surgery under general anesthesia. The incidence of intraoperative hypothermia, the primary outcome, was significantly higher in patients who received remimazolam than in those who received inhalation anesthetics. The effect size (Cohen's h = 0.637 for the primary outcome) indicates that the magnitude of the difference is substantial enough to be considered not only statistically significant but also clinically meaningful. Additionally, the administration of remimazolam resulted in a lower temperature at the end of surgery and a higher incidence of using warming devices in the PACU than the administration of inhalation anesthetics did. Changes in HR during the first hour after anesthesia induction were consistently observed with remimazolam compared with inhalation anesthetics.

Research on temperature regulation with the use of remimazolam is limited, and findings are inconsistent 12, 13. A study on rabbits indicated that the use of remimazolam significantly reduced core temperature changes during shivering, suggesting its potential to prevent shivering in surgical or therapeutic hypothermic settings 15. However, recent human studies, including ours, have yielded varying results 12, 13. Specifically, in a comparison of propofol with remimazolam as induction agents for laparoscopic prostatectomy, no significant differences were found in the incidence of intraoperative hypothermia 13. In this study, propofol-remifentanil and remimazolam-remifentanil were administered for induction and maintenance, respectively. In contrast, a study examining remimazolam as a maintenance agent during gynecologic surgery with sevoflurane as a comparator revealed a significantly lower incidence of postoperative shivering with remimazolam 12. In this study, remimazolam was used for anesthesia induction in both groups, whereas maintenance drugs included desflurane-remifentanil and remimazolam-remifentanil. Various factors have contributed to the observed differences, including the type of drug, timing of injection, and variations in primary outcomes. Previous and current studies have suggested that the choice of drug administered during both the induction and maintenance phases may significantly influence hypothermia. Specifically, in a previous study focusing on prostate surgery, the use of remifentanil for anesthesia maintenance was noted in both groups. Remifentanil, an opioid known to compromise body temperature regulation, can have a sustained effect during surgery 13, 16, 17.

However, our study may have diverged because of the exclusive use of inhalation anesthetics in the control group. Desflurane, a common inhalation anesthetic, exerts dose-dependent effects on body temperature. Inhalation anesthetics exhibit a slight, nonlinear effect in reducing the vasoconstriction threshold at low concentrations, while intravenous anesthetics demonstrate a pronounced, linear effect in reducing the vasoconstriction threshold 11, 17-19. Although inhalation anesthetics significantly lower the threshold at concentrations above 1 MAC, our study, which used BIS for anesthetic-depth monitoring, included patients with concentrations below 1 MAC 5, 20. This may have resulted in a smaller than expected impact. Based on our research, it appears that inhalation anesthetics may be more effective than a combination of remimazolam and remifentanil as anesthetic maintenance agents in preventing a decrease in body temperature.

Despite a higher incidence of hypothermia and lower core temperature in the group receiving remimazolam, there were no discernible differences in thermal comfort scores, the occurrence of shivering, or the severity of shivering in the PACU between the two groups. The threshold for shivering is approximately 35.5 °C in an awake patient 21. In our study, the mean core temperature at the end of surgery was 36.2 °C in the IA group and 35.9 °C in the R group. According to our study protocol, forced-air warming (set at 43 °C) was initiated not only when shivering occurred, but also in cases where the patient's temperature dropped below 36 °C or when the patient reported feeling cold. Therefore, although the incidence of intraoperative hypothermia was higher and the core temperature at the end of surgery was lower in the R group, which resulted in greater application of warming devices, the occurrence of shivering would not have shown a difference between the two groups. In addition, this could be attributed to the possibility that remimazolam has a lower threshold for shivering and responds earlier 15. Although core temperature is useful, it does not provide information on heat content. Similarly to our study, several previous studies have also found differences in the incidence of hypothermia, but no discernible differences in the occurrence of shivering 22-24. However, the shivering experienced by the patients may have appeared later because the duration of action of remimazolam was longer than that of desflurane 12, 25.

The hemodynamic stability associated with remimazolam is a characteristic feature, as demonstrated in our study, with consistent results 26. Even in older patients, the use of remimazolam exhibited greater hemodynamic stability than did propofol during induction, possibly because of its superior ability to maintain systemic vascular resistance. In our study, the change in blood pressure from induction to 1 h thereafter did not differ significantly between patients who were administered desflurane versus those who were administered remimazolam. However, the HR stability with remimazolam administration aligns with that of previous research, suggesting the drug's potential utility in patients who are hemodynamically unstable or who may encounter issues related to an increased HR 27, 28.

Our study has several limitations. First, the influence of remifentanil and nitrous oxide cannot be disregarded because each group received a combination of drugs rather than a single agent. A study examining the thermoregulatory threshold of desflurane and total intravenous anesthesia (TIVA) revealed that TIVA using propofol and remifentanil more effectively preserved body temperature 19. Given the diversity of drugs used in our study, further research is essential to discern the individual effects of each drug on body temperature regulation. Second, body temperature in the PACU is typically measured from the tympanic membrane using an infrared thermometer, which may differ from the core temperature measured during surgery. However, the primary outcome of this study was intraoperative hypothermia, which was measured using the core temperature of the esophagus. Because the intraoperative core temperature was measured through the esophagus, it did not affect the primary and secondary outcomes. Third, because the difference between the core and skin temperatures was not measured, the vasoconstriction threshold could not be determined, and it is unknown how remimazolam affects vasoconstriction threshold. Determining the vasoconstriction threshold in a future study would be helpful to confirm the impact of remimazolam on thermoregulation and the development of intraoperative hypothermia. Finally, the type and dose of vasoactive drugs and other medications used that affect hemodynamics during surgery may have confounded the results. Although the percentage of vasoactive drug use did not differ between the two groups, this does not imply that the drugs used to maintain hemodynamics during surgery had no effect on the patient's body temperature. Therefore, the possibility of a confounding effect cannot be excluded.

In conclusion, the incidence of intraoperative hypothermia in patients who underwent endoscopic nasal surgery was higher with the administration of remimazolam than with that of desflurane for the maintenance of anesthesia. Additionally, remimazolam demonstrated potential utility in maintaining hemodynamics, as evidenced by the consistent HR observed up to 1 h after induction. This emphasizes the importance of close monitoring of body temperature and strategies to prevent hypothermia in patients receiving remimazolam during surgery, with the aim of reducing the potential complications associated with hypothermia. To enhance the robustness and generalizability of our findings, further research involving a broader range of surgical procedures and diverse patient populations would be helpful.

## Authorship

All the listed authors were involved in the drafting of the work, approved the final manuscript, and agreed to be accountable for all aspects of this work.

1. Sung-Ae Cho: This author helped with writing the manuscript and analyzing and interpreting the data.

2. Seok-Jin Lee, Woojin Kwon, Ji-Yoon Jung, Hwang-Ju You, Si-eun Yoon: These authors helped with the acquisition, analysis, and interpretation of the data.

3. Tae-Yun Sung: This author helped with the conception and design of the study, statistical analysis, and writing of the manuscript.

## Figures and Tables

**Table 1:** Demographic and intraoperative data

Variable	Inhalation anesthetics (n = 31)	Remimazolam (n = 33)	P
Age, years	46.0 [30.0; 53.5]	45.0 [30.0; 53.0]	0.925
Weight (kg)	70.5 ± 14.0	70.0 ± 12.0	0.891
BMI (kg/m2)	25.4 ± 4.1	24.8 ± 3.4	0.509
ASA physical status (I/II)	7/24	4/29	0.437
Duration of anesthesia, min	91.0 [77.5; 110.0]	90.0 [75.0; 106.0]	0.567
Amount of intravenous fluid, mL	300.0 [250.0; 500.0]	400.0 [300.0; 500.0]	0.114
Amount of irrigation fluid, mL	300.0 [200.0; 400.0]	300.0 [125.0; 350.0]	0.558
Estimated blood loss, mL	20.0 [10.5; 27.5]	13.0 [10.0; 25.0]	0.533
Ambient temperature at the beginning of surgery, °C	22.3 ± 1.0	22.1 ± 1.0	0.312
Ambient temperature at the end of surgery, °C	22.4 [22.0; 22.9]	22.4 [21.7; 22.7]	0.505
Baseline body temperature, °C	36.9 ± 0.4	36.9 ± 0.4	0.494
Baseline SBP, mmHg	141.839 ± 19.5	134.7 ± 14.2	0.099
Baseline HR, rate/min	77.1 ± 14.3	72.8 ± 12.1	0.200
Use of hemodynamic drugs			
Phenylephrine	2 (6.5%)	3 (9.0%)	0.999
Nicardipine	7 (22.6%)	4 (12.1%)	0.437
Ephedrine	17 (54.8%)	18 (54.5%)	0.999
Atropine	1 (3.2%)	0 (0%)	0.975
Esmolol	9 (29.0%)	3 (9.1%)	0.085

Data are expressed as mean ± standard deviation, median (interquartile range), number, or number (%). ASA: American Society of Anesthesiologists

**Table 2:** Temperature-related data in operating room and PACU

Variable	Inhalation anesthetics (n = 31)	Remimazolam (n = 33)	RR or MD (95% CI)	Effect size d or h	P
In operating room					
Intraoperative hypothermia	10 (32.3%)	21 (63.6%)	1.863 (1.116, 3.110)	0.637	0.014
Severity of hypothermia					>0.999
Mild (35.5-35.9 °C)	10 (100%)	20 (95.2%)	NA	0.283	
Moderate (35.0-35.4 °C)	0 (0%)	1 (4.8%)	NA	0.442	
Severe (34.5-34.9 °C)	0 (0%)	0 (0%)	NA	0.000	
Temperature at the end of surgery, °C	36.2 ± 0.3	35.9 ± 0.4	0.240 (0.071, 0.408)	0.845	0.006
In PACU					
Thermal comfort score	8.0 [5.0; 9.0]	8.0 [5.0; 10.0]	NA	0.112	0.639
Use of warming device	3 (9.7%)	11 (33.3%)	1.355 (1.037, 1.770)	0.597	0.047
Incidence of shivering	1 (3.2%)	2 (6.1%)		0.139	>0.999
Grade of shivering	0.0 [0.0; 0.0]	0.0 [0.0; 0.0]	NA	0.242	0.331
Use of meperidine	0 (0%)	0 (0%)	NA	0.000	>0.999

Data are expressed as mean ± standard deviation, median (interquartile range), number, or number (%). MD: mean difference; RR: relative risk; CI: confidence interval; PACU: postanesthetic care unit

**Table 3:** Postoperative data and adverse events

Variable	Inhalation anesthetics (n = 31)	Remimazolam (n = 33)	*P*
NRS score for pain	4.0 [2.0; 5.5]	4.0 [2.0; 5.0]	0.844
Use of fentanyl	6 (19.4%)	10 (30.3%)	0.312
Adverse events			
Nausea	1 (3.2%)	0 (0.0%)	0.975
Headache	1 (3.2%)	6 (18.2%)	0.105
Dizziness	0 (0%)	0 (0%)	>0.999
Dyspnea	0 (0%)	0 (0%)	>0.999
Desaturation	0 (0%)	0 (0%)	>0.999
Confusion	0 (0%)	0 (0%)	>0.999

Data are expressed as median (interquartile range), number, or number (%). NRS: numerical rating scale

## Abbreviations

ASA PS: American Society of Anesthesiologists physical status
PACU: post-anesthesia care unit
BIS: bispectral index
SBP: systolic blood pressure
HR: heart rate
MAC: minimum alveolar concentration
NRS: numerical rating scale
ANOVA: analysis of variance
RR: relative risk
CI: confidence interval
MD: mean difference
TIVA: total intravenous anesthesia
